# Aortic Dilatation on the Edge of Dissection—Do We Operate Too Late? The Ratio between Ascending and Descending Aorta DiameteR (RADAR)

**DOI:** 10.3390/jcm12134400

**Published:** 2023-06-29

**Authors:** Nerea Lopez Perez, Philippe Reymond, Mustafa Cikirikcioglu, Mathieu van Steenberghe, Tornike Sologashvili, Nicolas Murith, Thomas Perneger, Christoph Huber

**Affiliations:** 1 Cardiovascular Surgery Department, University Hospitals of Geneva (HUG), 1205 Geneva, Switzerland; 2 Clinical Research Center, University Hospitals of Geneva (HUG), 1205 Geneva, Switzerland

**Keywords:** cardiac surgery, ascending aorta dissection, ascending aorta aneurysm

## Abstract

(1) Background: There is a need for a novel surrogate marker to ease decision making when facing ascending aortic dilatation. In this article, we study the ratio between ascending and descending aorta diameters as a potential one. (2) Methods: Retrospective observational cohort study, including all the patients who underwent surgery for acute type A aorta dissection (aTAAD) between January 2014 and September 2020 at our center. A total of 50 patients were included. Clinical and demographic data were collected. The anatomical measurements were made including orthogonal maximal diameters of the ascending and descending aorta, post-dissection whole circumference length (post-wCL), post-dissection true lumen circumference length (post-tCL), and surface and sphericity indices of the ascending and descending aorta. Pre-dissection ascending aorta diameter (pre-AAD) and pre-dissection descending aorta diameter (pre-DAD) were calculated as well as the ratio between them and compared with reference values. (3) Results: Of the pre-AAD patients, 96% had smaller than the recommended 55 mm. The ratio between the descending and ascending aorta pre-dissection diameters was significantly smaller compared to the reference value (0.657 ± 0.125 versus 0.745 ± 0.016 with a mean difference of −0.088 and a *p* < 0.001). (4) Conclusions: The 55 mm threshold for aorta maximal diameter is an insufficient criterion when assessing the risk of dissection. The ratio between DAD and AAD is a parameter worthy of analysis as a tool to stratify the risk of dissection.

## 1. Introduction

Acute ascending aortic type A dissection (aTAAD) is a life-threatening condition with an increasing annual incidence of 3–6 cases/100,000, compared to the 2.9/100,000 cases in the 2000s [1]. It has a higher incidence in men and an overall in-hospital mortality around 60% for patients with medical management alone and 26% among those treated surgically [2,3]. This makes prevention a key point in the management of ascending aortic dilatation.

Based on the current American (AHA) and European (ESC) guidelines [4,5], prophylactic ascending aorta replacement surgery is generally indicated at an aneurysmal diameter equal or greater than 55 mm despite previous studies presenting evidence of aTAAD occurring at diameters below the recommended threshold. In 1996, the International Registry of Acute Aortic Dissections (IRAD) study already showed that most of the included patients with aTAAD had a diameter inferior to 55 mm [3].

In 2007, Pape et al. reported [6], after looking at the results of 591 aTAAD patients of the IRAD registry, that 59% of them had an AAD < 55 mm and 40% had an AAD < 50 mm. With this, they highlighted the need for new methods to identify patients at risk for dissection. Moreover, in a recent study, Tozzi et al. concluded that aTAAD occurred at a computed aortic diameter below 45 mm in 87.7% of their patients and hence reinforced the concept that diameter should be a part of a patient-based decision for preventive ascending aorta replacement procedures [7].

Since then, new proposals have emerged to stratify the risk of dissection, such as a smaller diameter threshold, a BSA-indexed diameter [8], or the use of the ascending aortic length [9], but none of them have achieved enough evidence for clinical use.

In order to examine our experience and explore a novel surrogate marker (RADAR) based on the ratio between the descending and ascending aortic diameters, we decided to conduct this study. The goal was to facilitate patient-tailored decision making rather than to apply the one-size-fits-all concept in ascending aortic dilatation.

## 2. Materials and Methods

### 2.1. Patient Selection

**Figure 1:**
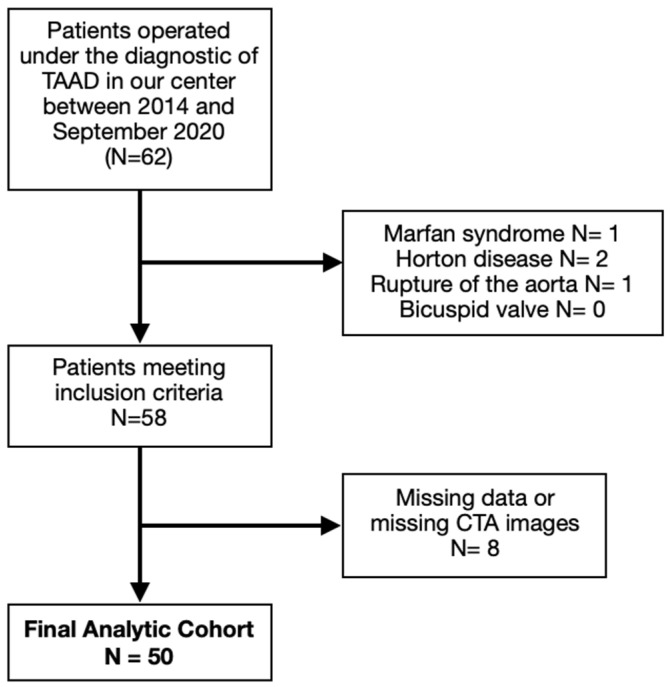
Flow chart diagram of patient selection. TAAD: thoracic ascending aortic dissection.

A retrospective observational study was conducted at our center with 62 patients who underwent surgery for aTAAD between January 2014 and September 2020. Patients with bicuspid aortic valve (verified with preoperative transesophageal echocardiography), connective tissue disorders, Horton disease, and traumatic or iatrogenic ascendant aortic dissection were excluded. Within this cohort, patients with non-available CT-scan images at admission or missing data were also excluded. A total of 50 patients were finally included (Figure 1).

### 2.2. Data Collection

Demographic and clinical variables, including cardiovascular risk factors, were collected retrospectively from medical charts and electronic medical records by physician review. The following parameters were recorded: age, gender, BSA, weight, height, arterial hypertension, and smoking and alcohol history. Data were also extracted from preoperative TTE images, including number of aortic valve cusps and degree of aortic valve insufficiency.

**Figure 2:**
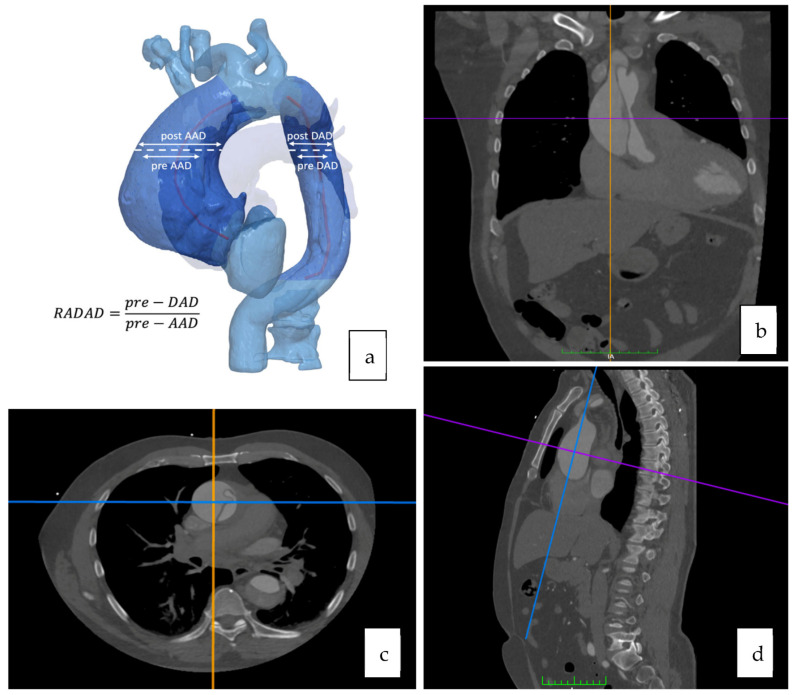
Imaging process and methodology. (**a**) Three-dimensional schematization. (**b**–**d**) Osirix© curved multiplane reformats, orthogonal plan (independently for AA and DA).

**Figure 3:**
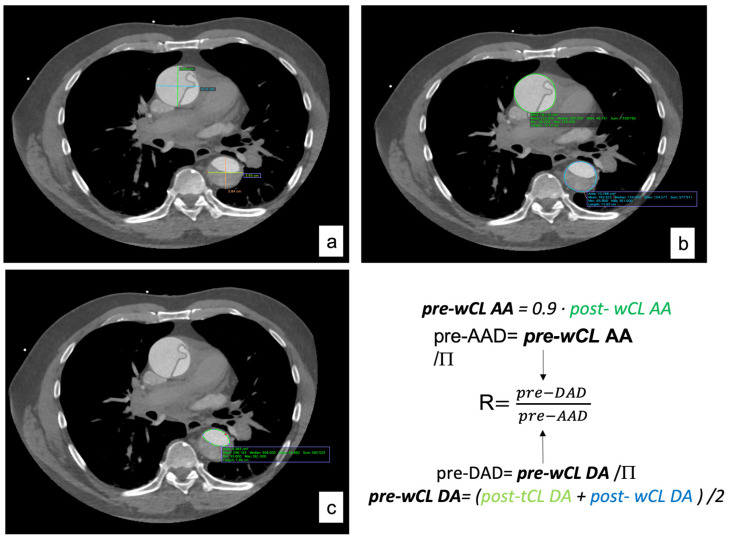
Imaging process and methodology. (**a**) Maximal diameters (AP and LL) of AA and DA. (**b**) AA post-wCL and area and DA post-wCL and area. (**c**) DA post-tCL and area.

The computer tomography angiography measurements were analyzed using curved multiplane reformats with the DICOM viewer OsiriX vd.12.01. Measures were manually taken in orthogonal planes (sagittal, axial, and coronal). A reference point to perform all the measures was determined at the level of the pulmonary artery bifurcation (Figure 2 and Figure 3).

The maximal diameter (including the false lumen) of the ascending and descending aorta was measured in two perpendicular planes (anteroposterior and medio-lateral) and the sphericity index (ratio between major and minor axis) was calculated.

The whole dissected aortic circumference length and surface were obtained. Finally, the circumference and surface of the true lumen were also recorded for both the ascending and the descending aorta.

To calculate the pre-dissected AAD, we extrapolated it from the whole circumference length (wCL) [pre-dissected AAD = pre-dissected wCL/P], which was obtained with the T. Yamauchi et al. equation [10].

[pre-wCL = 0.9 × post-wCL]

As for the descending aorta, the equation applied was the one proposed for clinical use by T. Yamauchi in his article from 2018 [11].

[pre-wCL = (post-tCL + post-wCL)/2]

The diameter was then extrapolated from the perimeter with the equation [pre-DAD = pre-dissected WCL/Π]. Once the maximal pre-dissected AAD and DAD were obtained, the ratio between them was computed.

### 2.3. Imagining Analysis

The pre-dissected AAD and DAD obtained were compared, with normal aorta diameters, defined by gender, age and BSA, as reported by Wolak et al. in their 2008 study [12].

They assessed the normal limits of ascending and descending aortic dimensions via non-contrast gated cardiac CT, adjusted to age, gender and BSA, in a large, low-risk population of subjects undergoing coronary artery calcium scanning.

Additionally, the ratio computed between pre-dissected DAD and AAD was compared individually according to gender, sex, and BSA to a reference ratio extrapolated from the Wolak et al. database [12].

The data analysis was performed by the epidemiology and statistical department at our center using the statistical software SPSS version 25. Parametric tests were used for comparison between the observed and the reference diameter and ratio values.

## 3. Results

A total of 50 patients were included. The mean age was 65.8 ± 11.68 years and the male gender was predominant, at 64%. The mean weight and height were 78.84 ± 16.56 Kgs and 171.58 ± 8.40 cm, respectively. The mean BSA was 1.91 ± 0.21 Kg/m^2^. Arterial hypertension was present in 62% (31) of the patients. Aortic valve insufficiency was identified via TTE or TOE in 38% (19) of patients. Smoking and alcohol drinking habits were observed in 32% (16) and 34% (17) of the sample, respectively. The total mortality (30-days) was 16% (8) with a 10% (5) intraoperative mortality (Table 1).

Ninety-six percent (48) of the patients had a pre-AAD smaller than the current 55 mm cut-off value. The mean calculated pre-AAD was 40.87 ± 7.95 mm compared to a gender and BSA matched general population reference value of 34.24 ± 1.61 (*p* < 0.001). Twenty percent of the patients did not present an extension of the dissection into the descending aorta. The mean calculated pre-DAD was 26.41 ± 5.32 mm, not showing a statistically significant difference with the reference DAD value (*p*-value = 0.415) (Table 2). The sphericity indices of the ascending and descending aorta were 0.93 ± 0.06 and 0.91 ± 0.09, respectively.

**Figure 4:**
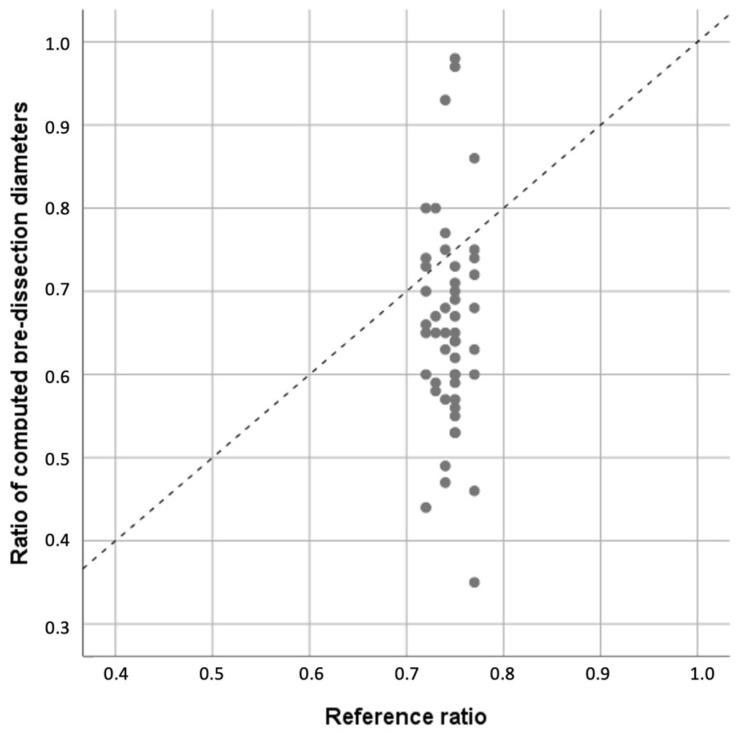
Ratio of computed pre-dissection diameters of the ascending and descending aorta in 50 patients as a function of reference ratio values from a general population (Wolak et al.) [12]. The dashed line is the identity line.

The ratio of the computed pre-ADD and pre-DAD was 0.66 ± 0.13. The reference ratio from a population-based study [12] was 0.74 ± 0.016. Their mean difference in ratios was −0.088 (95% confidence interval −0.124 to −0.051, *p* < 0.001), showing a significantly lower average compared with the reference values. The ascending aorta diameter was, on average, 1.5 times larger than the descending aorta diameter, as illustrated in Table 2 and Figure 4. The corresponding scatter plot illustrates the dispersion of the calculated pre-dissection values (Figure 4).

## 4. Discussion

Preventing aTAAD is still a major challenge. As the literature [6,7] and our results highlight, the currently recommended 55 mm threshold is insufficient to prevent aTAAD. We are facing a prevention paradox in which, thanks to operating on patients with an AAD larger than 55 mm, we barely see aTAAD in patients that exceed this cutoff. However, there is still a dissection risk gradient under the 55 mm threshold that we should not neglect, and hence new anatomic and radiologic criteria should be investigated. Eleftriades et al. [13] hypothesized the relative risk of aortic dissection at sub-surgical diameters and concluded that the risk under 45 mm is very low but that the risk increases by 6.3 when going over that value, recommending vigilance. They also called for further research for surgical decision making.

Alternative risk stratification tools and clinical surrogate markers have been proposed in the past few years such as, for example, the ascending aorta surface or the elongation of the ascending aorta [9,13,14].

Masri, A. et al. [8,15] described an increased mortality in those patients with an aortic cross-sectional area/height ratio above 10 cm^2^/m obtained using new three-dimensional technology. On another hand, Krüger et al. [9,16,17] found that an increase in the ascending aorta length in CTA was associated with a higher risk of aortic dissection. Based on their findings, the authors of the TAIPAN study in 2017 proposed a prophylactic aortic replacement for those patients with an ascending aortic length superior or equal to 12 cm and ascending aortic diameter between 45 and 54 mm [9]. This idea was also supported by Jinlin Wuu et al. [18] in a more recent study in 2019 in which they concluded that an elongation between 11.5 and 12 cm increases the probability of aortic adverse events (rupture, dissection, and death). However, evidence is yet not strong enough to clinically use thesenew landmarks.

It is widely acknowledged that the aortic diameter has an important interindividual variability, and hence a more patient-tailored approach is mandatory to assess the individual risk of dissection. A retrospective review of the CT scans of the patients we operated on for aTAAD shows that most of the patients presented with overall AAD below the recommended cut-off value, but also had strikingly large discrepancies between the AAD and the DAD.

The aorta remodels with growth, but the correlation between the ascending and descending aorta should remain stable. We therefore hypothesize that an AAD remarkably larger than the DAD translates to an abnormal remodeling and might increase the risk of dissection.

Our results are based on a sample of patients with remodeled aortas due to acute dissection, and hence the diameter observed in a post-dissection aorta does not correspond to the pre-dissection diameter. As Nakashima [19] described, the base for aortic dissection is the medial weakness caused by structural abnormalities in the elastic fibers. This idea was also supported by Roberts et al. [20], who evidenced a loss of the medial elastic fibers that compose the media in dissected aortas. For that reason, we must use modeled or calculated pre-dissection values.

Different authors have tried to estimate the pre-dissection diameter. Mansour et al. [21] concluded, after analyzing the AAD of 3400 patient prior to and post-dissection, that the pre-AAD is 7.65 mm smaller than the observed diameter after dissection. Moreover, a 7 mm smaller pre-AAA was used by Tozzi et al. to extrapolate the pre-AAD [7]. Due to high interindividual variability, we considered the equations proposed by Yamauchi et al. to calculate the pre-AAD and pre-DAD more accurately [10,11]. That being said, using the equations, we deducted a mean of 5.81 mm from the post-AAD.

Finally, based on the calculated-before-dissection AAD and DAA, there is a significant difference (*p*-value < 0.001) between the pre-dissection DAA/AAD and AAD/DAD ratios in our patients and the reference ratio from a population-based study. The main ratio of AAD/DAD that we obtained (1.58 ± 0.33) implies an ascending aorta diameter 1.5 times greater than the descending aorta diameter compared to the calculated reference value from the Wolak et al. database (1.34 ± 0.03).

### Limitations

Despite showing a statistically significant difference between the ratios (*p*-value < 0.05), our results are based on a small, mono-centric patient sample (*n* = 50), with observed diameters from already dissected ascending aortas. Furthermore, they were compared to a calculated reference value extrapolated from a population with normal aorta diameters instead of a population with aneurysmatic aortas, which would be a more interesting control.

Moreover, looking at the plot-chart (Figure 4), there is an important dispersion in the sample, with outliers with ratios close to 1 and ratios smaller than 0.4. This variability can be explained by, first, the fact that, once dissected, the aorta is remodeled entirely and hence, even using approved equations, the pre-dissected diameters can be over-estimated (ratios > 0.4) and under-estimated (ratios close to 1). It might also be explained by the variability in the extent of the dissection regarding the involvement of the descending aorta. In our sample, 20% of the patients had isolated ascending aorta dissection without descending progression.

## 5. Conclusions

The ascending aorta maximal diameter is an insufficient standalone criterion to assess dissection risk. Due to the limitations of our study, we cannot confirm the ratio between ascending and descending aorta diameters as an independent risk factor for dissection. That being said, a cut-off value of an ascending aortic diameter of 1.5 times the descending aortic diameter might be a patient-tailored surrogate marker for increased dissection risk. RADAR combined with other parameters such as elongation of the aorta, body surface indexed diameters, ulceration, and presence of a bicuspid valve might highly enhance guidance for dilated ascending aortas with diameters of less than 55 mm as a new risk stratification score. Further clinical investigation needs to be undertaken to confirm our results.

## Figures and Tables

**Table 1:** Demographic characteristics.

Demographic Characteristics (*n* = 50)	
Gender	
Male gender	64% (32)
Female gender	36% (18)
Arterial hypertension	62% (31)
Smoking habits	32% (16)
Alcohol drinking habit	34% (17)
Aortic insufficiency	38% (19)
Mean age (y.o.)	65.68 ± 11,68
Mean height (cm)	171.58 ± 8.40
Mean weight (Kg)	78.84 ± 16.56
Mean BSA (cm^2)^	1.91 ± 0.21
Total 30-day mortality	16% (8)
Peri-operative mortality	10% (5)

**Table 2:** Results of the mean imputed pre-AAD and pre-DAD obtained, the reference AAD and DAD values, and ratio between pre-DAD/pre-AAD obtained and reference. * Parametric test (t-student). SD: standard deviation, SE: standard error, Sig = *p* value (<0.005).

			Test Statistics *	
	Mean and SD	SE	t	Sig.
Ascending Aorta				
Imputed pre-AAD	40.87 ± 7.95	1.12		
Reference AAD	34.24 ± 1.61	0.23		
Pre-AAD and Ref-AAD	6.63 ± 7.99	1.13	5.86	*p* < 0.001
				
Descending Aorta				
Imputed pre-DAD	26.41 ± 5.32	0.75		
Reference DAD	25.45 ± 1.75	0.25		
Pre-DAD and Ref-DAD	0.96 ± 4.83	0.68	1.406	0.166
				
Ratio DAD/AAD				
Pre-D ratio	0.67 ± 0.19	0.023		
Ref-ratio	0.74 ± 0.02	0.003		
Pre-D ratio and Ref Ratio	(−)0.07 ±0.19	0.03	(−)2.624	0.012
Ratio AAD/DAD				
Pre-D ratio	1.58± 0.33	0.046		
Ref-ratio	1.34 ± 0.03	0.004		
Pre-D ratio and Ref Ratio	0.236 ± 0.33	0.047	5.041	*p* < 0.001

## Data Availability

The data underlying this article are available and will be shared on reasonable request to the corresponding author.

## Abbreviations

aTAAD	Acute type A aortic dissection
AA	Ascending aorta
AAD	Ascending aorta diameter
BSA	Body surface area in cm^2^
CTA	Computer tomography angiography
DA	Descending aorta
DAD	Descending aorta diameter
Pre-AAD	Pre-dissected ascending aorta diameter
Pre-DAD	Pre-dissected descending aorta diameter
Post-wCL	Post-dissected whole circumference length
Post-tCL	Post-dissected true lumen circumference length
RADAR	Ratio between ascending and descending aorta diameter (radium^2^)
TAAD	Type A aortic dissection
TTE	Transthoracic echocardiogram
wCL	Whole circumference length
